# Probing the Interaction of Human Serum Albumin with Norfloxacin in the Presence of High-Frequency Electromagnetic Fields: Fluorescence Spectroscopy and Circular Dichroism Investigations

**DOI:** 10.3390/molecules16129792

**Published:** 2011-11-25

**Authors:** Olga Azimi, Zahra Emami, Hanieh Salari, Jamshidkhan Chamani

**Affiliations:** 1 Department of Biology, Faculty of Sciences, Mashhad Branch, Islamic Azad University, Mashhad, 9175687119, Iran; Email: olga.azimi@gmail.com (O.A.); hnsalari@yahoo.com (H.S.); 2 Department of Physics, Faculty of Sciences, Mashhad Branch, Islamic Azad University, Mashhad, 9175687119, Iran; Email: Zahra_sh_emami@yahoo.com

**Keywords:** HSA, norfloxacin, spectroscopy, electromagnetic field, fluorescence quenching

## Abstract

The present study describes an investigation by fluorescence quenching, circular dichroism and UV-visible spectroscopy of the interaction between norfloxacin (NRF) and human serum albumin (HSA) in the presence of electromagnetic fields (EMFs). The results obtained from this study indicated that NRF had a strong ability to quench HSA at *λ*_ex_ = 280 nm. In addition, a slight blue shift occurred, which suggested that the microenvironment of the protein became more hydrophobic after addition of NRF. The interaction between the NRF and HSA, whether in the absence or presence of an EMF, was considered to be a static quenching mechanism. Moreover, synchronous fluorescence demonstrated that the microenvironment around Trp became modified. Data of HSA-NRF in the presence of EMFs between 1 Hz–1 MHz confirmed the results of quenching and blue shifts. Corresponding Stern-Volmer plots were also drawn and the resultant Ksv and kq values were compared. Moreover, the binding parameters, including the number of binding sites, the binding constant and the distance, r, between donor and acceptor, were calculated based on Förster’s non-radiative energy transfer theory. According to far and near UV-CD, the formation of the complex caused changes of the secondary and tertiary structures of HSA. The obtained results are significant for patients who are subjected to high-frequency radiation as this was found to reduce the affinity of NRF to HSA.

## 1. Introduction

There exist several reports on the effects of electromagnetic fields (EMFs) on proteins. The possibility that RF (Radio Frequency) radiation may cause changes in protein conformation and hence in biological properties has been revealed [1,2]. George *et al.* [3] studied citrate synthase unfolding under the effect of EMFs, and concluded that microwaves affected the protein conformation in the form of a direct interaction of the EMFs with the protein or its hydration water. In light of what happens to the biological systems when they are exposed to EMFs, there is some evidence of possible effects involving electron transfer reactions, as extensively reported by Blank and coworkers [4,5,6]. Nowadays, sonography, radio and wireless systems have become an inseparable part of human life. Sonography, or ultrasonography, uses sound waves in order to generate an image for the assessment and diagnosis of various medical conditions. Diagnostic medical sonographers direct high frequency sound waves into areas of the patient's body with the use of special equipment. As known, such technologies are always accompanied by propagation of EMFs in the environment. This has raised some controversial issues within the scientific community regarding the potential hazardous effects on human health [7], and it is therefore of importance to study the effects of EMFs on biological systems.

Protein-ligand binding plays a key role in the distribution and metabolism of organic and inorganic compounds in biological systems. One such extensively studied protein is HSA, the most abundant serum protein, which has many physiological and pharmacological functions [8,9]. However, the most significant one is to regulate plasma osmotic pressure between the blood and tissues. Another important function is to serve as a depot and transporting vehicle. As the latter, it works dominantly as a carrier transporting endogenous and exogenous compounds in the body such as fatty acids, hormones, bilirubin, drugs, and metal ions [10,11,12,13,14,15].

HSA is a single polypeptide with 585 amino acid residues in three homologous helical domains (I, II, III), 17 pairs of disulfide bridges and one free cysteine. Each of these three domains of the albumin monomer consists of two sub-domains (A and B). There is only one Trp located at position 214 along the chain, in sub-domain II A of HSA [11,12,13,14,15,16,17].

Information on the interaction of HSA with drugs can help us better understand the absorption and distribution of drugs [11]. Albumin is one of the longest known and probably the most studied of all proteins. Its manifold functions have attracted the interest of scientists and physicians for generations. Its applications are many, both in clinical medicine and in basic research [18].

**Scheme 1:**
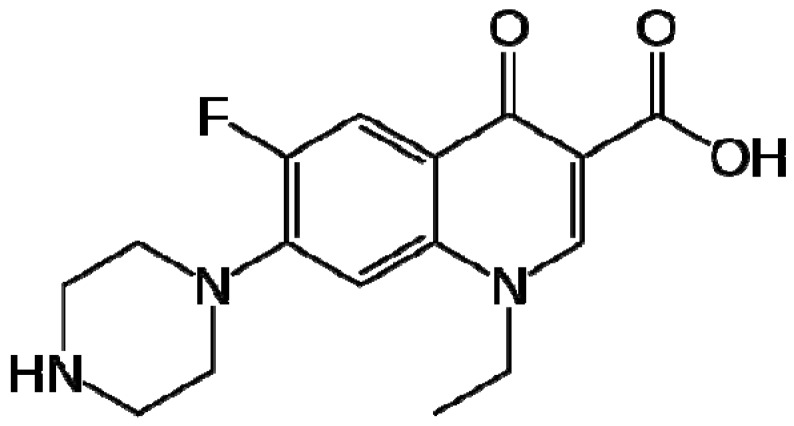
The chemical structure of norfloxacin.

Norfloxacin (NRF) is a fluoroquinolone antibiotic class which has bactericidal activity. The molecular weight of norfloxacin is 319.34 g/mol and its chemical name is 1-ethyl-6-fluoro-1,4-dihydro-4-oxo-7-(1-piperazinyl)-3-quinoline carboxylic acid [19]. Its structure is presented in Scheme 1. Norfloxacin (NRF) is an antibiotic that can eliminate bacteria that cause a variety of bacterialinfections. It works by stopping bacterial growth [20]. It inhibits bacterial deoxyribonucleic acid synthesis by hampering bacterial DNA gyrase and can therefore be used as a wide-spectrum antibiotic. Norfloxacin is used therapeutically to treat urinary and genital tract infections; and has also been utilized to treat prostatitis, bacterial gastroenteritis, gonorrhoeic urethritis, proctitis and cervicitis, in addition to its employment as a prophylactic for neutropenic patients [21]. Norfloxacin is very slightly soluble in water [22] and hence only 35%–45% of the drug administered orally is absorbed [23].

The aim of the present work was to investigate the effects of high-frequency electromagnetic fields on the interaction between norfloxacin and HSA, as well as on the structure and binding behavior of HSA as a drug carrier protein. Because of the importance of HSA as a drug transporter in blood, we have attempted to study the effect of radio waves of EMFs on the norfloxacin affinity and structure of HSA. The frequency used ranged from 1 Hz–1 MHz, and the waves thus included ultrasonic waves that are used for diagnosis of the internal part of human bodies.

## 2. Results and Discussion

### 2.1. Fluorescence Quenching of HSA by NRF and Quenching Mechanism

Fluorescence spectroscopy is a powerful and very sensitive technique for studying molecular interactions that involve proteins since it is a highly sensitive, rapid, and simple qualitative analysis technique for determining the binding of chemical compounds to HSA. Generally, the fluorescence of a protein is caused by three intrinsic fluorophores present in the protein, *i.e.*, the Trp, Tyr and Phe residues. Actually, the intrinsic fluorescence of many proteins is mainly contributed to by Trp alone. Fluorescence spectroscopy is often the method of choice for studying properties such as stability, hydrodynamics, kinetics, or ligand binding, because of its exquisite sensitivity [24]. These biochemical applications of quenching are due to the molecular interactions that result in quenching [25].

**Figure 1:**
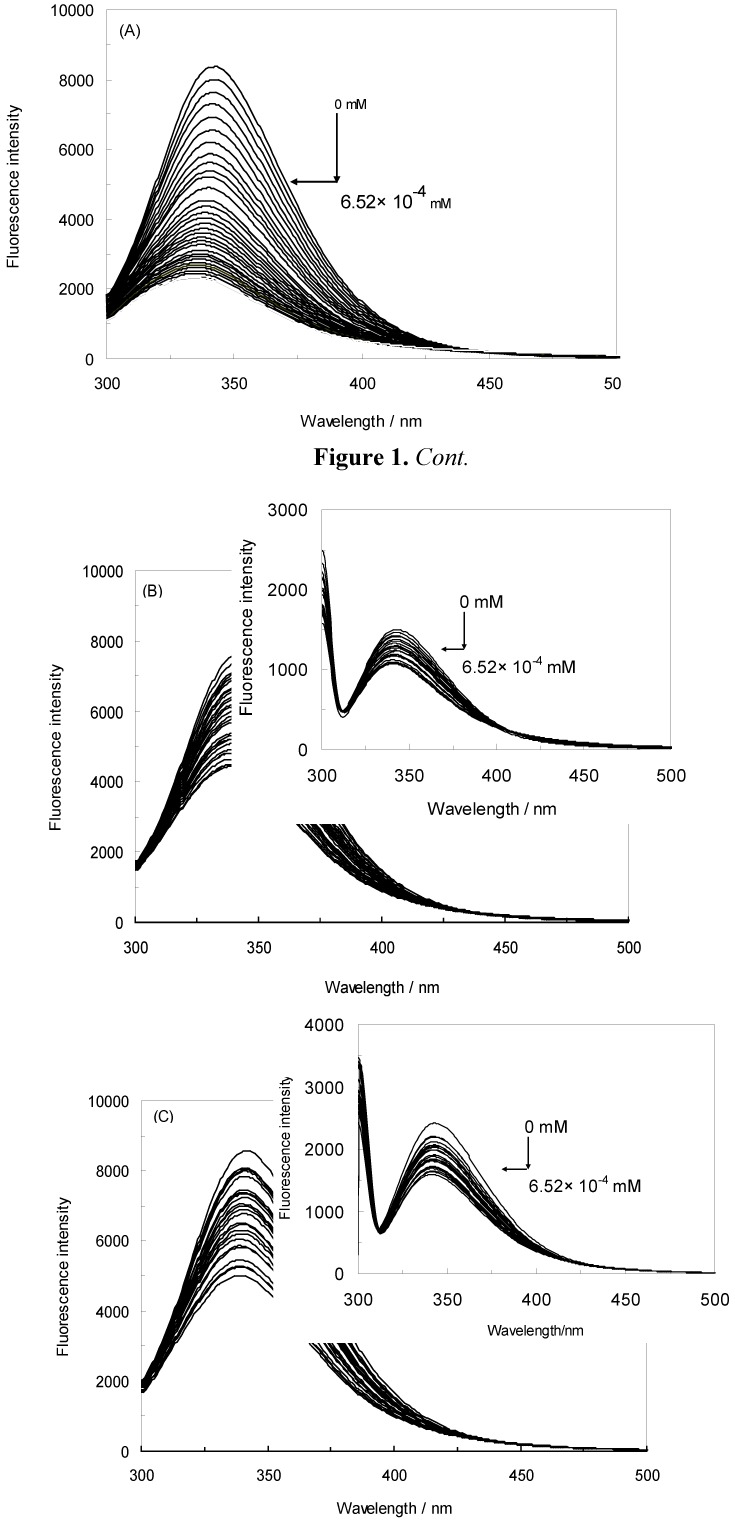
(**A**) Fluorescence emission spectra of the HSA-NRF system in the absence of an EMF; (**B**) Fluorescence emission spectra of the HSA-NRF system in the presence of an EMF at 100 HZ/2.4 mT; (**C**) Fluorescence emission spectra of HSA-NRF system in the presence of an EMF at 100 HZ/6.4 mT. The concentration of HSA was 4.5 × 10^−3^ mM and the NRF concentration was increased from (0 to 6.52 × 10^−4^) mM. T = 298 K; pH 7.40, λ_ex_ = 280 nm; (inset) λ_ex_ = 295 nm.

In this work, the binding between NRF and HSA under EMF exposure was investigated. The influence of norfloxacin on the HSA fluorescence intensity is shown in Figure 1A. The concentration of the HSA solution was constant at 4.52 × 10^−6^ M, whereas the concentration of NRF varied from 0 to 6.52 × 10^−4^ mM [26]. HSA was excited at 280 nm and 295 nm, and according to Figure 1A it revealed maximum fluorescence emission peaks at 343 nm and 343 nm, respectively. When the HSA solution was titrated with increasing amounts of NRF, its fluorescence intensities at 280 nm were found to be significantly decreased.

It can also be inferred from the spectra that NRF caused a slight blue shift from 343 nm to 335 nm of the maximum wavelength of the HSA fluorescence [11]. The strong quenching of the HSA fluorescence suggests that the chromophore of HSA was positioned in a more hydrophobic environment after addition of NRF [10]. The results further indicate that there were strong interactions between HSA and NRF [27]. The inset of Figure 1A illustrates the excitation at 295 nm. Just as at 280 nm, quenching and a blue shift occurred with addition of NRF. The maximum wavelength at 343 nm was shifted to 340 nm. When the 280 nm excitation wavelength was used, the fluorescence of HSA came from both Trp and Tyr residues whereas at 295 nm only the Trp residues were excited [28].

Figure 1B demonstrates the effect of NRF on the HSA fluorescence intensity in the presence of an electromagnetic field of 100 HZ/2.4 mT. It can be seen that the fluorescence intensity of HSA gradually decreased with increasing concentration of NRF in the presence of this EMF, indicating that NRF was bound to HSA. After the addition of NRF, the maximum wavelength of HSA shifted from 342 nm to 339 nm, which shows that the microenvironment around the chromophore became more hydrophobic [29]. The inset in Figure 1B shows a moderate decrease of the fluorescence intensity and a slight blue shift from 344 nm to 340 nm when the concentration of NRF is increased.

Figure 1C illustrates the effect of NRF on the HSA fluorescence intensity in presence of an EMF of 100 HZ/6.4 mT. It can be observed from the figure that there was a decrease in the fluorescence intensity caused by quenching, accompanied by a decrease of the wavelength emission maximum λ_max_ from 342 nm to 338 nm. This shift was reasonably attributed to an increased hydrophobicity of the region surrounding the chromophore. The inset in Figure 1C illustrates a modest decrease in fluorescence intensity and an insignificant blue shift from 342 nm to 341 nm after addition of NRF [29]. At excitation wavelengths of 280 nm and 295 nm, the fluorescence of HSA-NRF was thus quenched more in the absence of an EMF as opposed to in the presence of one. Consequently, the structure of HSA changed in the presence of an EMF.

To explain the data from the fluorescence quenching studies, one needs to determine what kind of interaction existed between protein and ligand. One way to distinguish the type of quenching is the use the Stern-Volmer equation and its modified counterpart. There are usually two types of quenching, classified into dynamic quenching and static quenching. Although the fluorophore can be quenched according to either of these types, a linear Stern-Volmer plot illustrates that a single class of fluorophores is equally accessible to the quencher [10,30]. For the sake of comparison, the quenching data were thus further analyzed by the well-known Stern-Volmer equation:

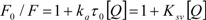
(1)
where F_0_ and F are the fluorescence intensities of HSA in the absence and presence of quencher, respectively, k_q_ is the quenching rate constant, K_sv_ is the Stern-Volmer quenching constant, τ_0_ is the average lifetime of the biomolecule without quencher (τ_0_ = 10^−8^ s [11]), and [Q] is the concentration of quencher [30]. We thus have:

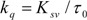
(2)
where k_q_ is the quenching rate constant.

**Figure 2:**
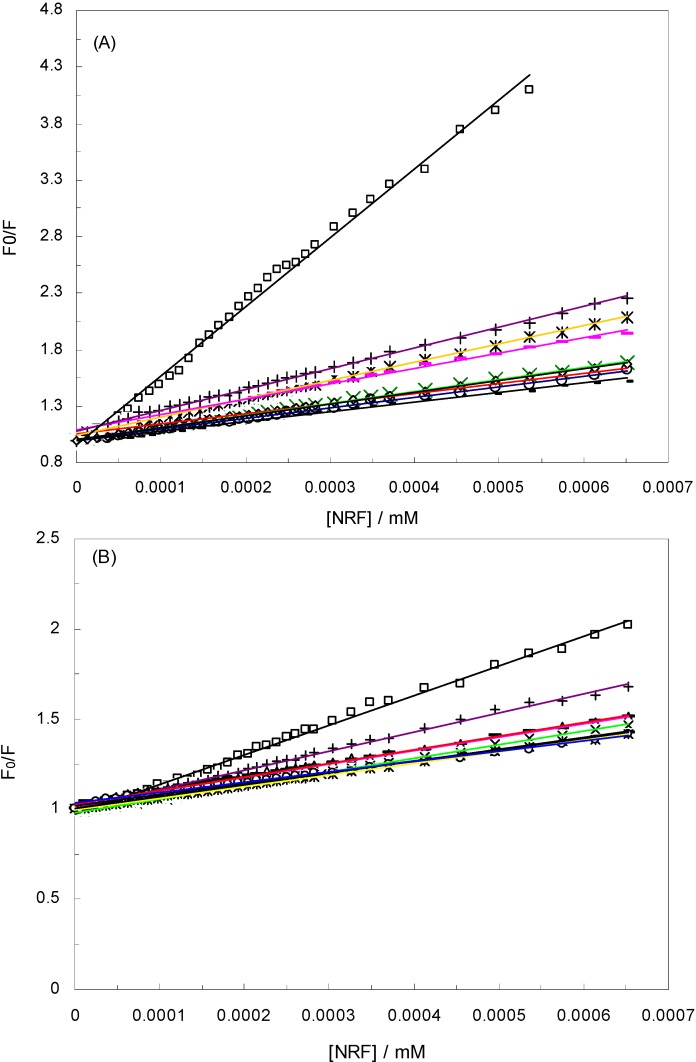
Stern-Volmer plots for the HSA-NRF systems in the presence and absence of EMFs. T = 298 K; pH 7.40, HSA-NRF (□), 100 Hz/2.4 mT (◊), 100 Hz/6.4 mT (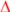), 1 KHz/0.98 mT (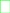), 1 KHz/1.9 mT (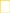), 10 KHz/0.17 mT (-), 10 KHz/63 μT 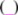, 100 KHz/39 μT (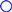), 1 MHz/59 μT (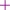), (**A**) at λ_ex_ = 280 nm, (**B**) at λ_ex_ = 295 nm.

Figure 2A shows the Stern-Volmer plots for the HSA-NRF systems in the presence different EMFs, at 280 nm, as well as in the absence of an EMF. The K_sv_ values in the absence and presence of different EMFs (100 Hz/2.4, 100 Hz/6.4, 1 KHz/0.98, 1 KHz/1.9, 10 KHz/0.17, 10 KHz/63, 100 KHz/39, 1 MHz/59) determined in Table 1. As can be seen, the affinity of HSA to NRF decreased approximately six fold in the presence of an EMF.

Moreover, the k_q_ values in the absence and presence of the EMFs were 6.12 × 10^14^, 1.04 × 10^14^, 0.9 × 10^14^, 1.05 × 10^14^, 1.61 × 10^14^, 0.86 × 10^14^, 1.36 × 10^14^, 0.94 × 10^14^ and 1.84 × 10^14^ L·mol^−1^·s^−1^, respectively. These values were much greater than the maximum collisional quenching constant (2.0 × 10^10^ L·mol^−1^·s^−1^). It was thus believed that static quenching was dominant in all systems [11]. Another way to establish this was by comparison of the k_q_ values of the protein excited at 280 nm and 295 nm which render it possible to estimate the participation of Trp and Tyr groups in the complex [31]. According to Table 1, in the presence of the EMF, the k_q_ value at *λ*_ex_ = 280 nm was greater than its counterpart at *λ*_ex_ = 295 nm. Finally, the Table also demonstrates that, in the absence of an EMF, the k_q_ and K_sv_ were greater than in the presence of one.

**Table 1:** Stern-Volmer quenching constants for the interaction of norfloxacin with HSA at various frequencies.

System	K_sv_ × 10^−6^/M^−1^ λ_ex_ = 280 nm	R λ_ex_ = 280 nm	K_sv_ × 10^−6^/M^−1^ λ_ex_ = 295 nm	R λ_ex_ = 295 nm	*f_a_* λ_ex_ = 295 nm	*f_a_* λ_ex_ = 280 nm
HSA-NRF	6.12	0.9956	1.65	0.996	2.05	0.7
HSA-NRF, 100 Hz (2.4 mT)	1.04	0.9978	0.63	0.9975	0.038	0.72
HSA-NRF, 100 Hz (6.4 mT)	0.9	0.9914	0.74	0.9971	0.28	0.31
HSA-NRF, 1 KHz (0.98 mT)	1.05	0.9984	0.76	0.9981	0.9	0.34
HSA-NRF, 1 KHz (1.9 mT)	1.61	0.998	0.65	0.9995	0.37	0.26
HAS-NRF, 10KHz (0.17 mT)	0.86	0.9905	0.65	0.9992	0.041	0.37
HSA-NRF, 10 KHz (63 μT)	1.36	0.9917	0.75	0.9977	0.37	0.21
HSA-NRF, 100 KHz (39 μT)	0.94	0.9963	0.58	0.9946	0.067	0.19
HSA-NRF, 1 MHz (59 μT)	1.84	0.9957	1.07	0.997	1.63	0.53

Figure 2B illustrates the Stern-Volmer plots for the HSA-NRF systems at 295 nm and at varying EMFs. According to this plot and the K_sv_ and k_q_ values from Table 1, it is obvious that regardless of the EMFs, the K_sv_ and k_q_ values were greater at the higher wavelength. Here, since we are dealing with a static quenching procedure, the modified Stern-Volmer equation can be used to analyze the data:

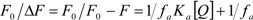
(3)
Where 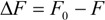 is the difference in fluorescence in the absence and presence of the quencher at concentration [*Q*], *f_a_* is the fraction of accessible fluorescence, and K_a_ is the effective quenching constant for the accessible fluorophores, which are analogous to associative binding constants for the quencher-acceptor system [26]. The plot of 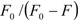 *versus* 1/[Q] is linear, with (1/fK) as the slope and 1/f as the intercept. The quenching constant K is a quotient of the intercept 1/*f* and slope (1/fK) [10]. (A plot of F_0_/∆F *versus* 1/[Q] yields *f_a_* ^−1^ as the intercept and (*f_a_* K_Q_)^−1^ as the slope. A y– intercept of *f_a_* ^−1^ may be understood intuitively).

Table 1 illustrates the fraction of accessible fluorescence value (*f_a_*) at 280 nm and 295 nm. It shows that the *f_a_* value of HSA-NRF in the absence of EMFs was higher than when an excitation wavelength of 295 nm was utilized. It can thus be concluded that not only did the Trp and Tyr play a major role in the interaction between HSA and NRF, it also became more efficient in the absence of an EMF according to the *f_a_* value in Table 1. When *f_a_* was equal to 1, all the fluorophore residues were accessible to the quencher. Consequently, a change in the value of *f_a_* signified that the fraction of fluorescent components accessible to the quencher was altered [32].

Since the fluorescence quenching was a result of a static quenching mechanism, the binding constant (K_a_) and the number of binding sites (n) can be determined by the following equation:

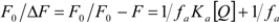
(4)
where the binding constant (K_A_) and the number of binding sites (n) are obtained through the ordinate and slope of the Hill curve of log 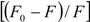 *versus* log 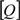 [33]. After analyzing the data with the Hill equation, the values of n and K_A_ could be obtained and they are listed in Table 2.

**Table 2:** Estimated values of the binding constant (Ka) and the possible number of binding sites (n) for binding of HSA with the NRF in the absence and presence of different EMFs at λex = 280 nm and λex = 295 nm.

System	K_a_ × 10^−6^/M^−1^ λ_ex_ = 280 nm	R λ_ex_ = 280 nm	n λ_ex_ = 280 nm	K_a_ × 10^−6^/M^−1^ λ_ex_ = 295 nm	R λ_ex_ = 295 nm	n λ_ex_ = 295 nm
HSA-NRF	22.06	0.9956	1.1589	8.252	0.996	1.2072
HSA-NRF, 100 Hz (2.4 mT)	6.43	0.9978	1.2154	0.21	0.9975	0.8561
HSA-NRF, 100 Hz (6.4 mT)	0.02	0.9914	0.5402	0.054	0.9971	0.6898
HSA-NRF, 1 KHz (0.98 mT)	0.66	0.9984	0.9409	0.42	0.9981	0.9233
HSA-NRF, 1 KHz (1.9 mT)	0.31	0.998	0.7889	1.307	0.9995	1.0866
HAS-NRF, 10 KHz (0.17 mT)	21.42	0.9905	1.4005	0.28	0.9992	0.8946
HSA-NRF, 10 KHz (63 μT)	0.11	0.9917	0.6698	0.086	0.9977	0.7185
HSA-NRF, 100 KHz (39 μT)	5.116	0.9963	1.2033	0.053	0.9946	0.6838
HSA-NRF, 1 MHz (59 μT)	0.4	0.9957	0.7908	0.033	0.997	0.5263

While the binding constant decreased significantly in the presence of an EMF, the number of binding sites remained almost constant [9,11]. At 280 nm and with a magnetic field of 10 KHz frequency and 0.17 mT, K_a_ did not become significantly altered, K_sv_ and k_q_ decreased, and n was almost stable. Moreover, it was found that the number of binding sites (n) was approximately 1 in the presence and absence of EMFs at 280 nm, suggesting that one molecule of HSA interacted with one molecule of the ligand.

**Figure 3:**
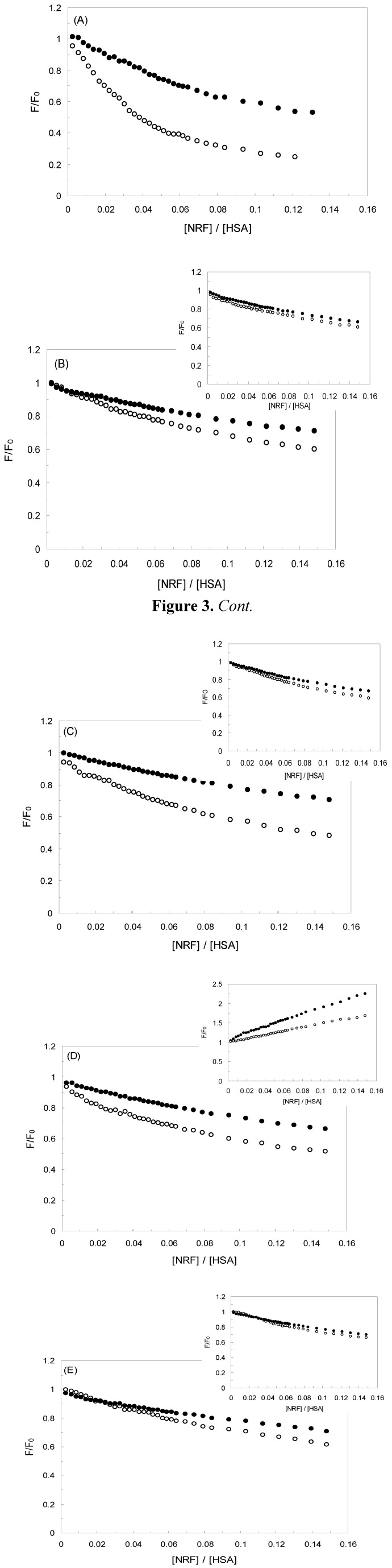
Comparison of the quenching curves for HSA-NRF in the absence and presence of EMFs. T=298K; pH 7.40. λ_ex_ = 280 nm (○); λ_ex_ = 295 nm (●). (**A**) HSA-NRF; (**B**) 100 Hz/2.4 mT, (inset) 100 Hz/6.4 mT; (**C**) 1 KHz/1.9 mT, (inset) 1 KHz/0.98 mT; (**D**) 10 KHz/63 μT,(inset) 10 KHz/0.17 mT; (**E**) 100 KHz/39 μT,(inset) 1 MHz/59 μt.

The quenching of HSA fluorescence by any ligand allows us to conclude that this ligand binds in the IIA sub-domain since albumin has only one Trp residue, located in this sub-domain. Additionally, a comparison of the quenching curves obtained at 280 nm and 295 nm excitation for HSA-NRF in the absence of EMFs is shown in Figure 3A. It can be clearly seen that they do not overlap and that the quenching curves at 295 nm were higher than those at 280 nm. This phenomenon shows that in the interaction of NRF with HSA, both the Trp and Tyr groups took part.

Figures 3 (B and E) further demonstrate quenching curves obtained at 280 nm and 295 nm excitation wavelength for HSA-NRF in the presence of EMFs. From Figure 3 (B-inset), it can be clearly seen that there is no overlap of the quenching curves of HSA 100 Hz/6.4 mT, pointing at participation of both the Trp and Tyr groups. At 100 Hz/2.4 mT, on the other hand, an overlap was visible at a concentration of NRF of 0.02 at the decreased field intensity and the quenching curves of HSA excited at 295 nm were slightly higher than those of HSA excited at 280 nm. These observations show that, in the interaction of NRF with HSA in the presence of 100 Hz/2.4 mT, only Trp took part up to a certain concentration of NRF after which both the Trp and Tyr groups participated. It can be concluded that when both Trp and Tyr were induced, the NRF molecules approached Trp and Tyr in HSA.

Similarly, Figure 3C shows that the quenching curves did not overlap whereas there was an overlap at 1 KHz/0.98 mT up to an NRF concentration of 0.03. This indicated that, with a decreased field intensity (such as 1 KHz/0.98 mT), NRF was located closer to Trp 214 than at 1 KHz/1.9 mT. As can be seen in Figures 3 (D and E), there was no overlap for any of the frequencies with a decreased field intensity, and the quenching curves of HSA excited at 295 nm were slightly higher than their counterparts excited at 280 nm. An exception to this trend can be seen in the inset of Figure 3E, where the quenching curves of HSA excited at 280 nm were slightly higher than those excited at 295 nm. Generally speaking, these figures may suggest that in the presence of EMFs, the structure of HSA changed and the role of Trp became clear.

### 2.2. Conformation Investigation by Synchronous Fluorescence

Synchronous fluorescence spectroscopy was introduced by Lloyd in 1971 [34]. The technique provides information about the molecular environment in the vicinity of the chromophore molecules and has several advantages, such as sensitivity, spectral simplification, spectral bandwidth reduction and the possibility to avoid different perturbing effects. It involves simultaneous scanning of the excitation and emission monochromators while maintaining a constant wavelength interval. It is a useful method to study the environment of amino acid residues by measuring the possible shift in wavelength emission maximum λ_max_, *i.e.*, the shift in position of the emission maximum corresponding to changes of the polarity around the chromophore molecule [35,36]. When the D-value (Δλ) between the excitation and emission wavelengths is set to 15 or 60 nm, the synchronous fluorescence can provide the characteristic information of respectively the Trp or Tyr residues in a protein [10,31].

**Figure 4:**
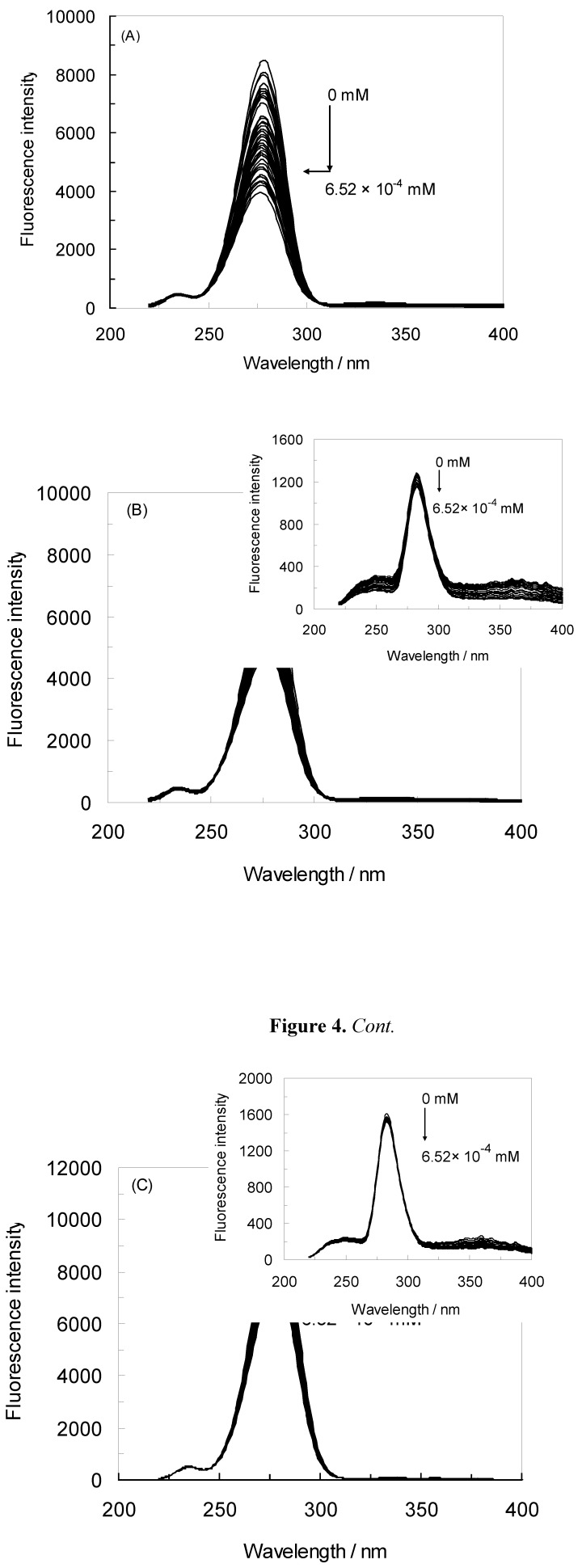
The synchronous fluorescence spectroscopy of HSA-NRF in the absence and presence of EMF, T = 298 K; pH 7.40. (**A**) The HSA-NRF system in the absence of an EMFfor Δλ = 60; (inset) Δλ = 15; (**B**) the HSA-NRF system in the presence of an EMF at 100 Hz/2.4 mT Δλ = 60; (inset) Δλ = 15; (**C**) the HSA-NRF system in the presence of an EMF at 100 Hz/6.4 mT Δλ = 60; (inset) Δλ = 15.

The synchronous fluorescence spectroscopy of HSA-NRF in the absence and presence of EMFs is shown in Figure 4. As can be seen, there were three different conditions corresponding to the absence of an EMF and the presence of two types of EMFs, *i.e.*, 100 Hz/2.4 mT and 100 Hz/6.4 mT. The synchronous fluorescence spectroscopy of HSA-NRF in the absence of an EMF is shown in Figure 4A. It can be seen that with an increasing concentration of NRF, the fluorescence of the Tyr residues was weak and the position of maximum emission wavelength did not shift when Δλ was 15 nm. Moreover, the fluorescence of the Trp residues was strong and the maximum emission wavelength shifted slightly toward shorter wavelengths, *i.e.*, a faint blue shift of approximately 1 nm, when Δλ was 60 nm. This suggests that the microenvironment around the Trp residue became little more hydrophobic when affected by NRF binding [10,37].

It is apparent from Figure 4B that the maximum emission wavelength of the Trp residue demonstrated a slight blue shift of about 1 nm and that the fluorescence moderately decreased with an increasing concentration of NRF in the presence of an EMF of 100 Hz/2.4 mT and Δλ = 60. This suggests that the polarity around the Trp residues decreased and that the hydrophobicity increased. Furthermore, there occurred no shift in the maximum emission wavelength when Δλ was 15 nm [10,37,38].

Figure 4C illustrates that the maximum emission wavelength of the Trp residues displayed a small blue shift of about 1 nm and that the fluorescence slightly decreased when increasing the concentration of NRF in the presence of an EMF of 100 Hz/6.4 mT and at Δλ = 60. However, no shift was observed when Δλ was 15 nm*.* By comparing these figures, it could be inferred that the conformation of HSA changed more in the absence of an EMF with increasing concentrations of NRF. Moreover, the fluorescence intensity presented a modest decrease when increasing the field intensity at Δλ = 60. It can also be mentioned that the microenvironment around the Trp residues became more hydrophobic when adding NRF at Δλ = 60 irrespective of other conditions.

**Figure 5:**
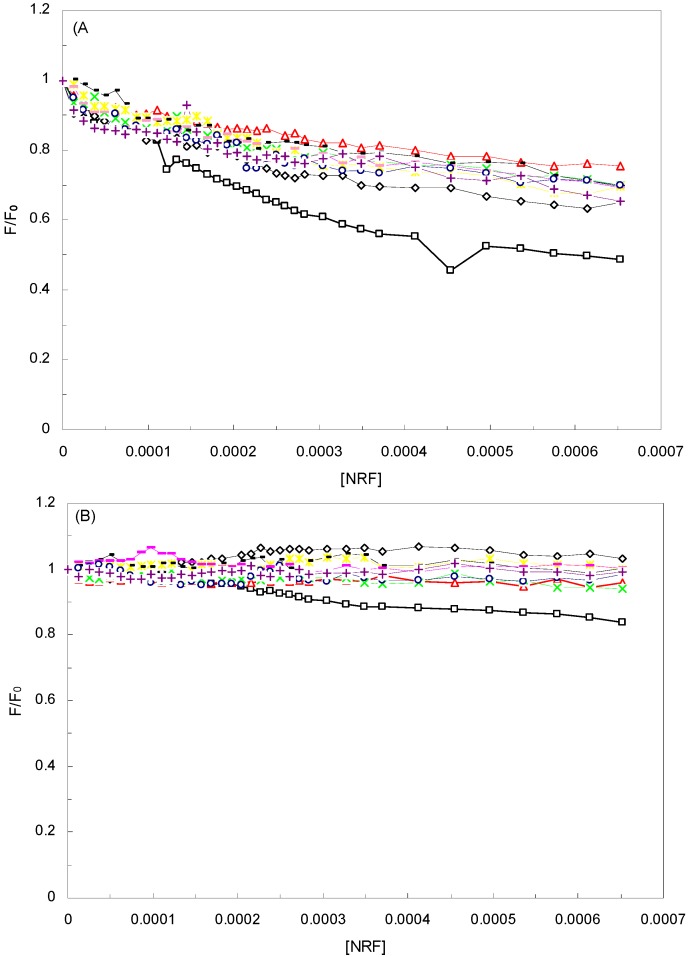
Synchronous fluorescence spectra of the quenching of HSA by NRF in the absence and presence of EMFs, T = 298K; pH 7.40. HSA-NRF(□), 100 Hz/2.4 mT(◊), 100 Hz/6.4 mT(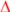), 1 KHz/0.98 mT(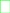), 1 KHz/1.9 mT(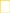), 10 KHz/0.17 mT (-), 10 KHz/63 μT 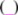, 100 KHz/39 μT (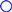), 1 MHz/59 μT (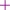), **(A)** in Δλ = 60. **(B)** Δλ = 15.

To explore the structural change of HSA when adding NRF and affected by an EMF, we measured the curves of F/F_0_ *versus* [Q] [Figures 5(A, B)] for the HSA-NRF system in the absence and presence of EMFs at various concentrations of the drug (at Δλ = 60 and Δλ = 15 nm). According to the figure, the slope of HSA-NRF in the absence of an EMF was higher than in the presence of EMFs when Δλ was 15 nm or 60 nm. This indicated that, in the absence of an EMF, the interaction between HSA and NRF was stronger than in the presence of one. By comparing the slopes of HSA-NRF at Δλ = (15, 60) nm, the slope at Δλ = 60 nm was higher from which can be deduced that the conformation of HSA and the polarity around the Trp residues had changed (data not shown). In addition, it was also determined from the figure that there was a significant contribution of the Trp residues to the fluorescence of HSA at Δλ = 60 nm, which indicated that NRF was closer to the Trp residues than to the Tyr residues at theinterface [36]. It can consequently be concluded that, during the binding process of NRF with HSA in the absence of an EMF, NRF affected the micro-environment of the Trp residue more than in thepresence of an EMF.

### 2.3. Resonance Light Scattering

Resonance light scattering (RLS) is an elastic scattering phenomenon that was first introduced and established by Pasternack *et al.* and its first application for analytical use was developed by Huang *et al*. [39]. It is often used to study of the aggregation and assembly of biological macromolecules by means of an ordinary fluorescence spectrometer. RLS is an extremely sensitive and selective technique for monitoring molecular assemblies [40]. In recent years, the method has been developed for the determination of proteins [41]. It can be combined with other techniques such as absorption, fluorescence and CD spectroscopies, and can compensate for the drawbacks of spectrophotometric and fluorometric measurements [42]. By scanning both the excitation and emission monochromators of a common spectrofluorometer with Δλ = 0 nm, RLS spectra can be recorded [43], and have proven to be useful when investigating the aggregation of small molecules as well as the long-range assembly of organic dyes on biological templates [43,44].

**Figure 6:**
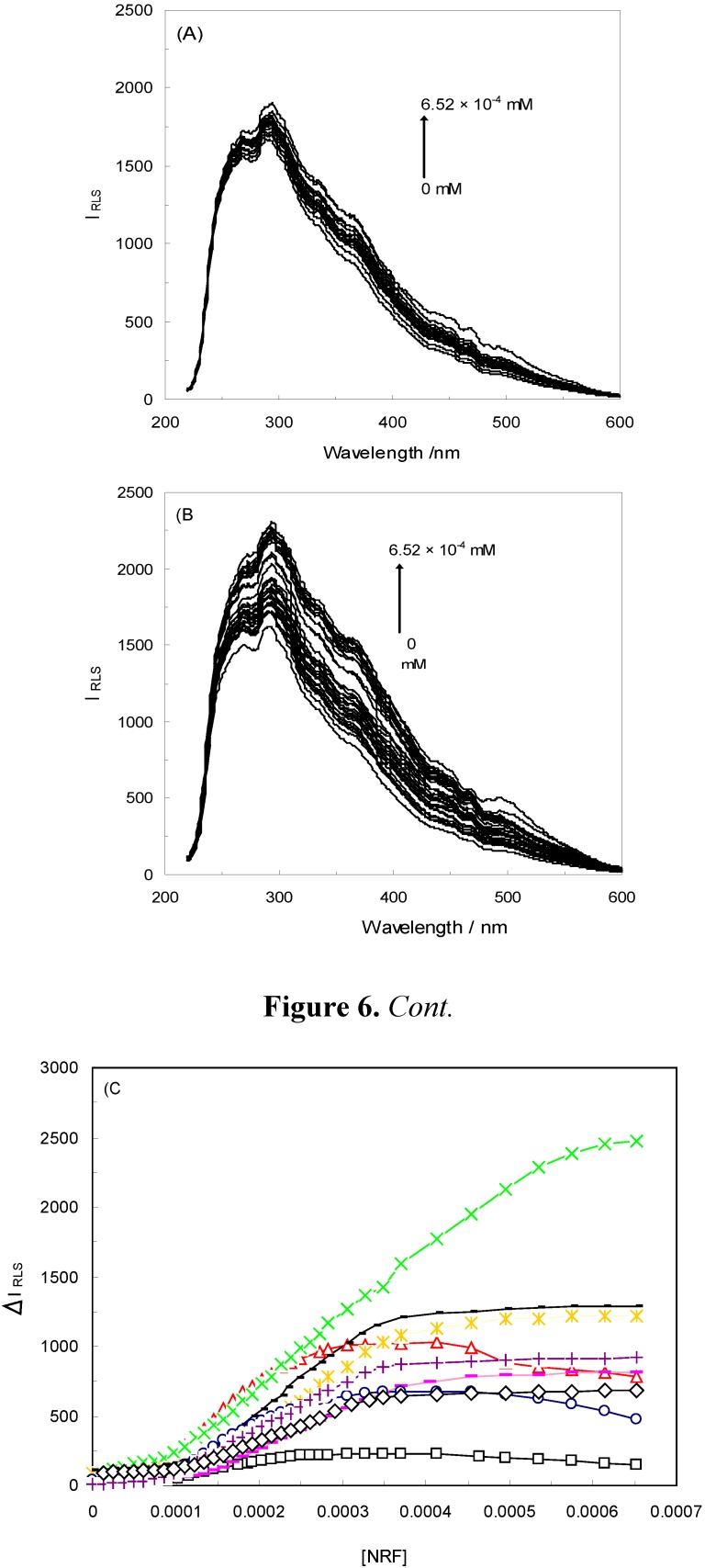
(**A**) The RLS spectra of HSA-NRF in the absence and presence of EMFs. The concentration of HSA was 4.5 × 10^−3^ mM and the NRF concentration was increased from (0 to 6.52 × 10^−4^) mM. T = 298 K; pH 7.40. The HSA-NRF system in the absence of an EMF; (**B**) HSA-NRF in the presence of an EMF (100 Hz/2.4 mT); (**C**) the curves of ∆I _RLS_ *versus* NRF in the absence and presence of EMFs for HSA-NRF(□), 100 Hz/2.4 mT(◊), 100 Hz/6.4 mT(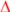), 1 KHz/0.98 mT(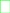), 1 KHz/1.9 mT(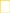), 10 KHz/0.17 mT(-), 10 KHz/63 μT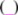, 100 KHz/39 μT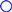 1 MHz/59 μT (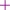).

Figure 6A shows the RLS spectrum of HSA in the presence of NRF. As can be seen, when adding NRF to HSA, the RLS intensity was increased, leading to the conclusion that an interaction had occurred between HSA and NRF. The reasons behind this increased RLS intensity were: (i) that larger particles were produced; (ii) that the self-aggregation of HSA gave rise to micelles; and (iii) that a ligand–protein complex was formed.

Figure 6B illustrates an RLS spectrum of HSA-NRF in the presence of an EMF (100 Hz/2.4mT). Similarly to the above-mentioned results, a modest increase in RLS intensity could be seen, which was proportional to the molecular weight of the proteins [14]. By comparing Figures 6(A and B), it was suggested that the RLS intensity of HSA-NRF in the presence of EMFs was higher than in the absence of one. The presence of EMFs was thus believed to give rise to more aggregated complexes of HSA-NRF.

Figure 6C portrays the analysis of the HSA-NRF system in the absence and presence of EMFs. The RLS intensity of the HSA-NRF systems slightly increased with an augmentation of the drug concentration and average-sized increments were seen in the presence of EMFs. It could thus to be concluded that an interaction occurred between HSA and NRF in the absence and presence of EMFs and that larger particles were produced, which led to the enhancement in RLS signals. In addition, as can be observed in Figure 6C, NRF aggregated on the HSA when the NRF concentration reached approximately 1 × 10^−5^ mM in both conditions; this was the critical induced aggregation concentration of the drug (C_CIAC_) [45].

As is illustrated in Figure 6C, the smallest ∆I _RLS_, which can be expressed as ∆I_RLS_ = I_RLS_ − I_0RLS_, where I_RLS_ and I_0RLS_ are the RLS intensities of the systems in the presence and absence of drug, respectively [36], belonged to HSA-NRF and the greatest corresponded to 1 KHz/0.98 mT. This shows that the presence of an EMF influenced the ∆I _RLS_. The results further prove that EMFs can induce protein aggregation and even the formation of precipitate. EMFs thus cause a behavioral change of the interaction between HSA and NRF.

### 2.4. Red Edge Excitation Shift (Theory and Application)

The red edge excitation shift (REES) is one of the effects that is observed when a polar fluorophore is placed in motionally restricted media such as a very viscous solutions or condensed phases where the dipolar relaxation time for the solvent shell around a fluorophore is comparable to or longer than its fluorescence lifetime [46]. Generally, the emission spectrum of a fluorophore is independent of the excitation wavelength and as such this principle is often used as an indication of the purity and/or homogeneity of a fluorescent sample. Thus, for most fluorophores in fluid solvents, the emission spectra are independent of the excitation wavelength and solvent viscosity [47]. Since the REES is observed only under conditions of restricted mobility, it has been used as a potential tool to estimate the fluorophore (both intrinsic and extrinsic) environment in organized biological assemblies such as membranes, micelles, and proteins [48].

Table 3 shows the REES values of HSA-NRF in the absence and presence of different EMFs. The differences seen amongst the REES values of these systems presumably reflect the different Trp environments. It is apparent from Table 3 that the REES values of HSA-NRF in the absence of an EMF (~7 nm) were greater than the corresponding values in the presence of EMFs. According to Table 1, it is evident that the emission maximum was shifted towards longer wavelengths (EERS) as the excitation wavelength was moved towards the longer wavelength side (REE). A similar behavior has been observed by others [49]. The REES increased in the order HSA-NRF > 1 KHz (1.9 mT), 10 KHz (0.17 mT), 1 MHz (59 μT) > 100 Hz (2.4 mT and 6.4 mT), 1 KHz (0.98 mT), 10 KHz (63 μT), 100 KHz (39 μT). Therefore, although the NRF decreased the mobility of Trp, the mobility was increased by the presence of EMFs.

**Table 3:** The REES values of HSA-NRF in the absence and presence of different EMFs.

System	NRF-HSA / 1:10
HSA-NRF	7
HSA-NRF, 100 Hz (2.4 mT)	2
HSA-NRF, 100 Hz (6.4 mT)	2
HSA-NRF,1 KHz (0.98 mT)	2
HSA-NRF, 1 KHz (1.9 mT)	4
HSA-NRF, 10 KHz (0.17 mT)	4
HSA-NRF, 10 KHz (63 μT)	2
HSA-NRF, 100 KHz (39 μT)	2
HSA-NRF, 1 MHz (59 μT)	4

### 2.5. Second Derivative Fluorescence Spectroscopy

Second derivative fluorescence spectroscopy is a sensitive and reliable technique for monitoring and characterizing the transitions that take place in the environments of aromatic amino acids (mainly tryptophans) in proteins. One advantage of using this technique lies in the possibility of monitoring processes in proteins, which involve relatively small changes in the Trp environments but which may not be clearly visible in the fluorescence spectra. Another advantage resides in the independence of the derivative spectra with respect to the turbidity of the samples, rendering corrections for light scattering unnecessary. A third advantage of using derivative methods in fluorescence spectroscopy is the selective excitation of Trp, which makes it much easier to separate effects due solely to this residue from those due to the combined effects of Trp and Tyr, as in absorption spectroscopy [50].

**Figure 7:**
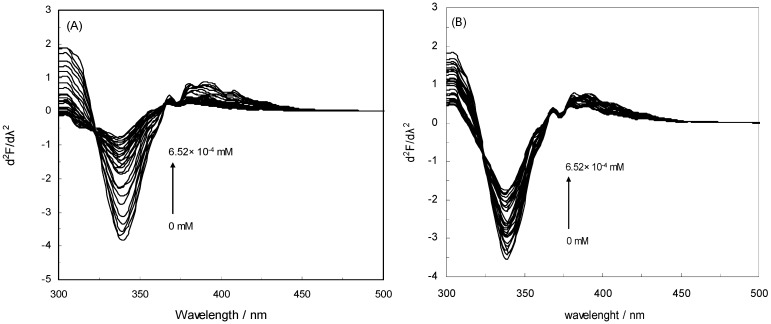
Second derivative fluorescence of HSA-NRF in the absence and presence of EMFs. (**A**) The HSA-NRF system in the absence of an EMF; (**B**) HSA-NRF in the presence of an EMF (100 Hz/2.4 mT).

In this study, the maximum emission wavelength of Trp in HSA was blue-shifted from 343 nm to 340 nm which indicates that the single Trp residue of HSA (Trp 214) was positioned in a more hydrophobic environment after complex formation with NRF in the absence of an EMF (inset Figure 1A). It is apparent from the inset of Figure 1B that there occurred a slight blue shift from 344 nm to 340 nm implying that Trp 214 was placed in a more hydrophobic environment after complex formation with NRF also in the presence of EMFs. Thus, to better understand the effect of NRF on the Trp of HSA, the second derivative fluorescence of the titration of NRF to HSA in the absence and presence of EMFs was analyzed, and the results are presented in Figure 7 (A and B) and Table 4. As can be seen, the second derivative spectrum (Figure 7A) demonstrated one minimum at about 339 nm, which suggests that the Trp of HSA was located in a more hydrophobic environment. Also Figure 7B showed a minimum at 340 nm, pointing at the same conclusion. The H values obtained from second derivative fluorescence spectroscopy determine the hydrophobicity score of protein upon interaction with ligand. Table 4 shows the H values of HSA-NRF in the presence of EMF at special molar fraction (1:10). It determines that the various EMF show the different behavior of interaction between NRF and HSA.

### 2.6. Energy Transfer from HSA to Drugs

According to Förster’s non-radiative energy transfer theory, the rate of energy transfer depends on: (i) the relative orientation of the donor and acceptor dipoles; (ii) the extent of overlap of the emission spectrum of the donor with the absorption spectrum of the acceptor; and (iii) the acceptor being close enough to the donor with a maximum distance of 7 nm [51].

**Figure 8:**
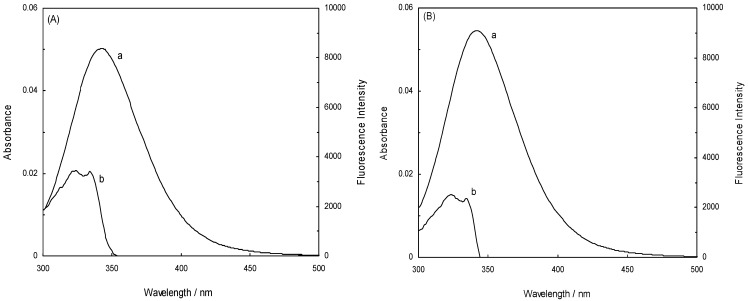
Spectral overlap of the fluorescence spectra (curves a) of HSA-NRF (**A**) and HSA-NRF at 1 MHz/59 μT (**B**) with the absorption spectrum (curves b) of NRF.

The overlapping of the UV absorption spectrum of Norfloxacin with the fluorescence emission spectrum of HSA in the absence and presence of EMFs is shown in Figure 8(A, B). The efficiency of energy transfer, E, is described by the following equation:

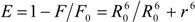
(5)
Here, F and F_0_ are the fluorescence intensities of HSA in the presence and absence of NRF, respectively, and r is the distance between acceptor and donor. R_0_ is the critical distance when the transfer efficiency is 50% according to:

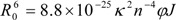
(6)
Where 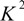 is the spatial orientation factor of the dipole, n is the refractive index of the medium, 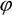 is the fluorescence quantum yield of the donor, n is the average refractive index of the medium, J is the overlap integral of the fluorescence emission spectrum of the donor and the absorption spectrum of the acceptor, (Figure 8). J is given by:

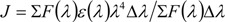
(7)
where F (λ) is the fluorescence intensity of the fluorescence donor at wavelength λ, and 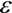 (λ) is the molar absorption coefficient of the acceptor at wavelength λ [11,31].

The distance between norfloxacin and amino acids in HSA was 1.98 nm in the absence of an EMF, whereas it was 3.11 nm for amino acids in HSA in the presence of EMFs, as can be seen in Table 4. It can furthermore be observed that these values decreased with increasing frequencies and that all of them were lower than 7 nm in the interaction between NRF and HSA. This is in accord with the conditions of Förster’s non-radiative energy transfer theory [35]. Moreover, these results suggest a static quenching mechanism in the interaction between the drugs and HSA in the absence and presence of EMFs [36,52]. The results illustrated that, in the presence of the EMFs, the distance between the drug and HSA decreased.

**Table 4:** The H value and the distance, r, between donor and acceptor of HSA with NRF in the absence and presence of different of EMFs.

System	H /1:10	r/ nm
HSA-NRF	7	1.98
HSA-NRF, 100Hz (2.4 mT)	2	2.03
HSA-NRF, 100Hz (6.4 mT)	2	2.08
HSA-NRF, 1KHz (0.98 mT)	2	2.27
HSA-NRF, 1KHz (1.9 mT)	4	2.33
HAS-NRF, 10KHz (0.17 mT)	4	2.51
HSA-NRF, 10KHz (63 μT)	2	2.57
HSA-NRF, 100KHz (39 μT)	2	2.62
HSA-NRF, 1MHz (59 μT)	4	3.11

### 3.7. Circular Dichroism and the Conformational Analysis

Circular dichroism (CD) spectroscopy is a powerful method in structural biology and has been used to examine proteins, polypeptides, and peptide structures [53]. The method has been proven to be sufficiently simple, reliable, and, in many circumstances, invaluable for a rapid determination of protein structure or the monitoring of conformational changes. Typically, the CD spectra of proteins are recorded in the far-UV region (180–250 nm) and the near-UV region (250–320 nm). An advantage of the CD technique in studies of proteins is that complementary structural information can be obtained from a number of spectral regions. The far-UV CD spectrum is directly related to the protein’s secondary structure and near-UV CD is characteristic of the tertiary protein structure [54,55]. The types of information which can be obtained from CD studies of proteins thus include: (i) the secondary structural composition (% α-helix, β-sheet, turns, *etc.*) from the peptide bond region; (ii) the tertiary structural fingerprint; (iii) the integrity of cofactor binding sites; (iv) conformational changes in the protein; and (v) protein folding [56]. 

The data was expressed as a molar residue ellipticity [θ], defined as [θ] = 100 θ_obs_ / cl, where θ_obs_ is the observed ellipticity in degrees, c is the concentration in mol residue cm^−3^, and l is the length of the light path in cm. CD spectra were recorded with a time constant of 4 s, a 2-nm bandwidth, a scan rate of 5 nm min^−1^ and a 1-mm path length cell from 250 to 190 nm. The data was signal-averaged over at least five scans, and the baseline was corrected by subtracting a buffer spectrum. The rotatory contributions of a protein can be determined as X = *f*_H_X_H_ + *f*_β_X_β_ + *f*_R_X_R_ where X is either the ellipticity or the rotation at any wavelength, *f* is the fractions of the helix (*f*_H_), beta form (*f*_β_) and unordered form (*f*_R_); the sum of *f* is equal to unity and each *f* is greater than or equal to zero. The thus determined CD for the helix, beta and random forms can be conversely used to estimate the secondary structure of any protein with X at several wavelengths for the same equation. The α-helical content (*f*_H_) was estimated from the ellipticity value at 222 nm ([θ]_222_) according to the following:

*f*_H_ = −([θ]_222_ + 2340 / 30300)

Secondary structural elements were calculated and confirmed how the protein structure changed. The results are listed in Table 5. According to the data, the secondary structure of free HSA consisted of ~53.97% α-helix, ~18.31% β-sheet, ~13.48% turn and ~14.25% unordered coil. The results in Table 5 demonstrate that the α-helix percentage decreased gradually, from 53.9% in free HSA to 53.17% with addition of NRF, whereas the content of unordered coil increased from 14.25% to 15.83%. This indicates a certain degree of HSA structurally destabilize.

**Table 5:** Secondary structural analysis of the HSA-NRF systems from CD data in the absence and presence of EMFs.

System	α-helix %	β-sheet %	Turn %	Unordered coil %
HSA	53.97	18.31	13.48	14.25
HSA-NRF	53.17	17.78	13.22	15.83
HSA-NRF, 100 Hz (2.4 mT)	53.06	17.61	13.17	16.16
HSA-NRF, 100 Hz (6.4 mT)	53.01	17.57	13.13	16.29
HSA-NRF, 1 KHz (0.98 mT)	52.93	17.44	13.03	16.6
HSA-NRF, 1 KHz (1.9 mT)	52.88	17.37	12.81	16.94
HAS-NRF,10 KHz (0.17 mT)	52.78	17.31	12.75	17.16
HSA-NRF, 10 KHz (63 μT)	52.76	17.31	12.72	17.21
HSA-NRF, 100 KHz (39 μT)	52.43	17.11	12.41	18.05
HSA-NRF, 1 MHz (59 μT)	51.77	16.59	12.27	19.37

**Figure 9:**
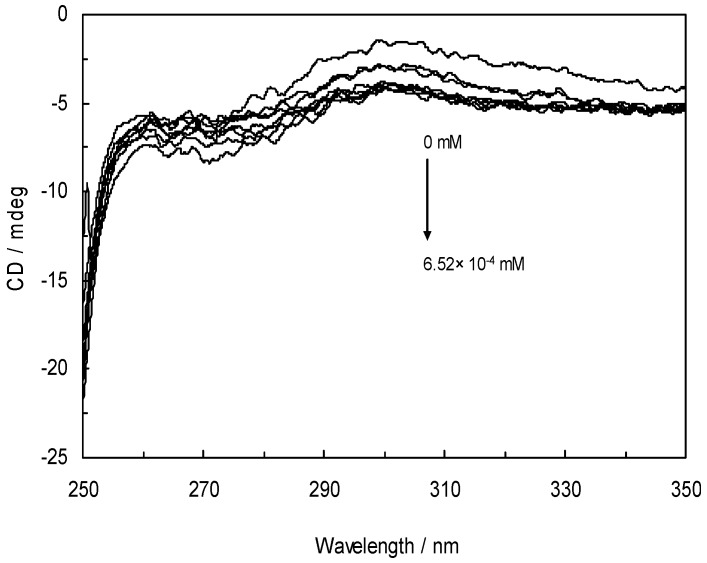
Near-UV CD spectra of HSA-NRF in the absence and presence of EMFs.

It is further noticeable from Table 5 that the α-helix percentage decreased gradually in the presence of EMFs of increasing frequencies. Generally, as is apparent from these results, the binding of NRF to HSA in the absence and presence of EMFs causes a conformational change of the protein, with the loss of α-helix stability. Figure 9 shows the changes in HSA tertiary structure upon NRF addition in the absence and presence of EMFs. Near-UV CD spectra of Trp in the protein are highly sensitive to interactions between nearby groups. Therefore, near-UV CD spectra of Trp potentially hold valuable conformational information about the protein [57]. Figure 9 demonstrates a gradual decrease in peak intensity when increasing the NRF concentration in the presence of EMFs, indicating that the tertiary structure of HSA was denatured.

## 3. Experimental

### 3.1. Apparatus

**Scheme 2:**
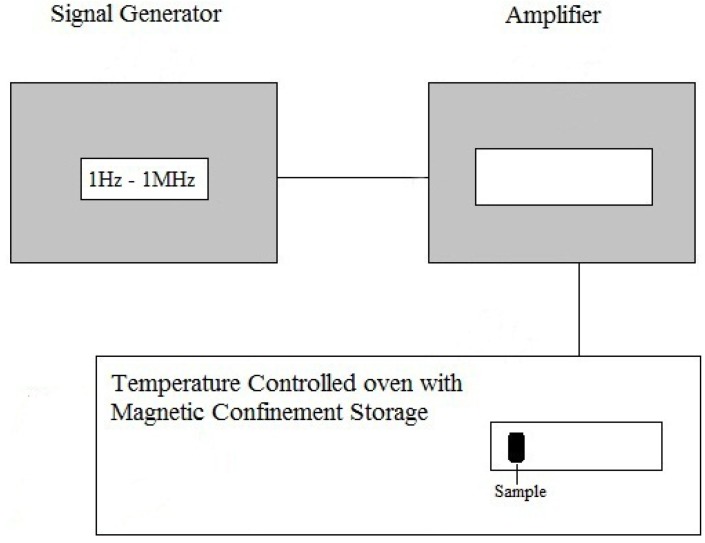
Block diagram for exposing the HSA-NRF samples.

A home-made electromagnetic field generator was used to expose the matter to EMFs. The instrument was equipped with a cell-holder with a controlled temperature and the frequency was ranged between 1 Hz–1 MHz as radio waves. The introduced magnetic fields in this apparatus were obtained from Ampere's law with the electric current in a coil in ac form. The EMF frequencies were selected by a signal generator attached to the equipment. The temperature of the experiments was controlled with an oven connected to the apparatus. The fluctuation of the EMF of the apparatus was beyond the limit of calculation. The set-up can be seen in Scheme 2.

UV/vis spectra were collected at room temperature on a double-beam V-630 spectrophotometer (Jasco, Tokio, Japan) in 1.0-cm quartz cells. The slit width was set at 5 nm and the wavelength range was 200–500 nm. All fluorescence measurements were carried out on an F-2500 spectrophotometer (Hitachi, Japan) equipped with 1.0-cm quartz cells and a thermostat bath with xenon lamp light. The excitation wavelengths were set to 280 nm and 295 nm. The excitation and emission slit widths were 5 nm. The scan speed was 1200 nm/min. The photo multiplier tube (PMT) voltage was 700 V. Fluorescence intensities were corrected for inner filter and dilution effects before analysis on the binding and quenching data. The synchronous fluorescence spectra were obtained by simultaneously scanning the excitation and emission monochromators with Δλ = 15 nm and with Δλ = 60 nm, respectively.

RLS spectra were recorded while simultaneously scanning the excitation and emission spectra from 280 to 600 nm with Δλ = 0 nm and with a slit width of 5.0 nm. This method has been proven to be able to determine the aggregation of small molecules as well as the long-range assembly of drugs on biological templates.

Circular dichroism (CD) was carried out as far-UV CD and near-UV CD experiments on a Jasco-815 spectropolarimeter equipped with a Jasco 2-syringe titrator. The scanning speed was 20 nm/min from 190 to 250 nm and 250 to 350 nm. Dry nitrogen gas was used to purge the equipment before and during the course of the measurements. The bandwidth was 1 nm and the scanning speed was 200 nm/min. The CD samples were prepared with a fixed concentration of HSA (0.03%) and varied drug concentrations resulting in equal total volumes. The instrument was calibrated with ammonium d-10-camphorsulfunic acid. The results are expressed as the mean residue defined as [θ] = 100 × θ_obsd_ / (LC), where θ_obsd_ is the observed ellipticity in degrees, C is the concentration in residue mol cm^−3^, and L is the length of the light path in cm. All pH measurements were performed with a Metrohm digital pH-meter (Metrohm, Berlin, Germany).

### 3.2. Reagents

HSA (fatty acid free, 90%), NRF and potassium phosphate were all purchased from Sigma-Aldrich Co. (St. Louis, MO. USA) and were used as supplied without further purification. The protein was dissolved in 50-mM phosphate buffer solutions at pH 7.4 and the stock solution was kept at 4 °C.

### 3.3. Procedures

NRF was dissolved in 50-mM potassium phosphate buffer at pH 7.4 and diluted to 0.05 mM corresponding to low usage dose concentrations. A digital pH-meter (Metrohm) was used for the pH adjustment. The HSA solution was added in order to make up 2 mL (4.52 × 10^−6^ M), and the range of the drug (NRF) solutions was gradually titrated manually into the cell using a micro-injector. It should be noted that after each injection, the cell was placed in the electromagnetic field generator. The reaction time was investigated and the results showed that 3 min was enough for the stabilization. The fluorescence spectra were then measured at an excitation wavelength of 280 nm and 295 nm and an emission wavelength of 290–600 nm. The UV/vis absorbance spectra of NRF were recorded at 291 nm at room temperature. The far-UV and near-UV CD spectra of NRF were read and spectral scanning curves were recorded under the same conditions.

## 4. Conclusions

The effects of EMFs on the structure and binding behavior of HSA to NRF were studied at physiological pH and temperature. By combining results obtained with intrinsic, second-derivative, synchronous fluorescence spectroscopies, REES, FRET, RLS as well as far-UV and near-UV CD, it was shown that EMFs changed the interaction between HSA and NRF. The processes that gave rise to fluorescence quenching included: excited-state reactions, energy transfer, complex formation and static quenching. The results pointed at the conformation of HSA becoming altered upon interaction with NRF in the presence of EMFs.

Moreover, by comparing the quenching of HSA fluorescence in the complex of HSA-NRF, and by analyzing the quenching constant values, it was found that EMF could alter the affinity of NRF to HSA. In fact, the affinity was decreased almost six fold. Furthermore, EMF could strengthen the NRF-induced conformational changes in HSA. The fluorescence quenching mechanism for HSA through NRF binding was thus static under both conditions for all systems.

The distance (r) between NRF and HSA was evaluated according to Förster’s theory of energy transfer. Aggregation of NRF/HSA complexes in the presence of EMFs was determined through RLS. The EMFs increased the mobility of Trp by REES. Alterations in the micro-environment of the aromatic residues were also observed by near UV-CD, second derivative fluorescence spectroscopy and synchronous fluorescence analysis.

According to the present study, precautions should be taken when patients who take NRF are exposed to radiation from wireless systems and ultrasonography. This is based on the fact that EMFs can reduce the affinity of NRF to HSA, which in turn may affect the distribution and pharmacological activity of the drug.
